# Data for the optimization of conditions for meat species identification using ultra-fast multiplex direct-convection PCR

**DOI:** 10.1016/j.dib.2017.11.004

**Published:** 2017-11-04

**Authors:** Kyung-Young Song, Hyun Jin Hwang, Jeong Hee Kim

**Affiliations:** a R&D center, Ahram Biosystems Inc., Seoul 133-120, Republic of Korea; b Department of Life and Nanopharmaceutical Sciences, Graduate School, Kyung Hee University, Seoul 130-701, Republic of Korea; c Department of Oral Biochemistry and Molecular Biology, Kyung Hee University, Seoul 130-701, Republic of Korea

**Keywords:** DNA-based, Multiplex, Meat species, Convection PCR

## Abstract

This article contains data related to the research article entitled “Ultra-fast DNA-based multiplex convection PCR method for meat species identification with possible on-site applications” (Song et al., 2017 [1]). Direct PCR that does not require prior DNA extraction is critical for ultra-fast molecular detection of meat species. We successfully acquired DNA by swab sampling in Taq DNA polymerase buffer. To reduce DNA sample preparation time, proteinase K incubation (0.2 μg/mL) and heat inactivation times were decreased to 10 min and 1 min, respectively. The analysis of swabbed DNA samples from mixed meat could differentiate meat species within the mixed sample. The swabbed DNA samples could be diluted 100 times without losing detection sensitivity.

**Specifications Table**

**Table:** 

Subject area	*Biology*
More specific subject area	*Biotechnology*
Type of data	*Table, figures*
How data was acquired	*DNA swab sampling and direct-convection PCR using Palm PCR G2-12, Ahram Biosystems, Inc., Korea*
Data format	*Analyzed*
Experimental factors	*Primers were designed using mitochondrial cytochrome b (Cyt b) gene sequences of three species (beef, lamb, and pork), obtained from the GenBank database. Meat samples To avoid cross-contamination between the meats, each piece of meat was cut with a clean and sterilized scalpel and the inner surfaces were used for sample preparation.*
Experimental features	*Direct-convection PCR*
Data source location	*N/A*
Data accessibility	*With this article*
Related research article	Song, K.-Y., Hwang, H.J., and Kim, J. H. Ultra-fast DNA-based multiplex convection PCR method for meat species identification with possible on-site applications, Food Chem. (2017) 229:341–346.

**Value of the data**

• Data presented here provide optimized conditions of a multiplex direct-convection PCR method for on-site detection of meat species.
• Taq DNA polymerase buffer was sufficient for sample extraction from swabs.
• Proteinase K incubation and heat inactivation time could be reduced to 11 min, with the total meat identification time of approximately 57 min.
• Multiplex direct-convection PCR was sensitive and robust enough to detect meat species in mixed meat samples.

## 1 Data

**Fig. 1:**
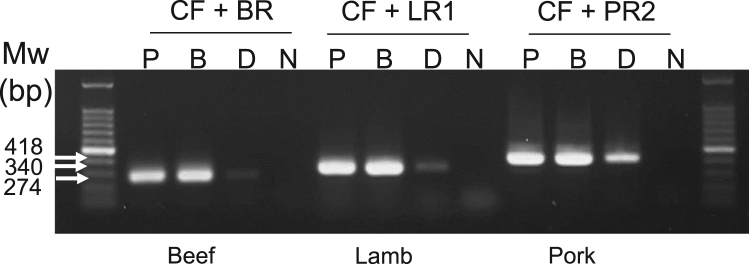
Meat identification in swabbed samples in different buffers. Samples were collected with sterile swabs that were then rinsed in phosphate buffered saline (PBS, lane P), *PalmTaq* Buffer (lane B), or distilled water (lane D). An aliquot of the solution was used in convection PCR. Species-specific primer sets (CF and BR, LR1, or PR2) were used. N, no template control; Mw, molecular weight marker.

**Fig. 2:**
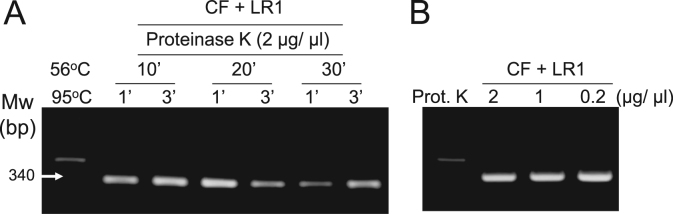
Optimization of proteinase K incubation and heat inactivation times for direct-convection PCR. (A) Proteinase K (2 μg/μL) treatment time was reduced from 30 to 20 and 10 min. Following each proteinase K treatment, 1 and 3 min heat inactivation times were tested. (B) Proteinase K concentration was reduced from 2 to 1 and 0.5 μg/μL. In (A) and (B), lamb meat was used as the sample, with lamb-specific primers (CF and LR1). Mw, molecular weight marker.

**Fig. 3:**
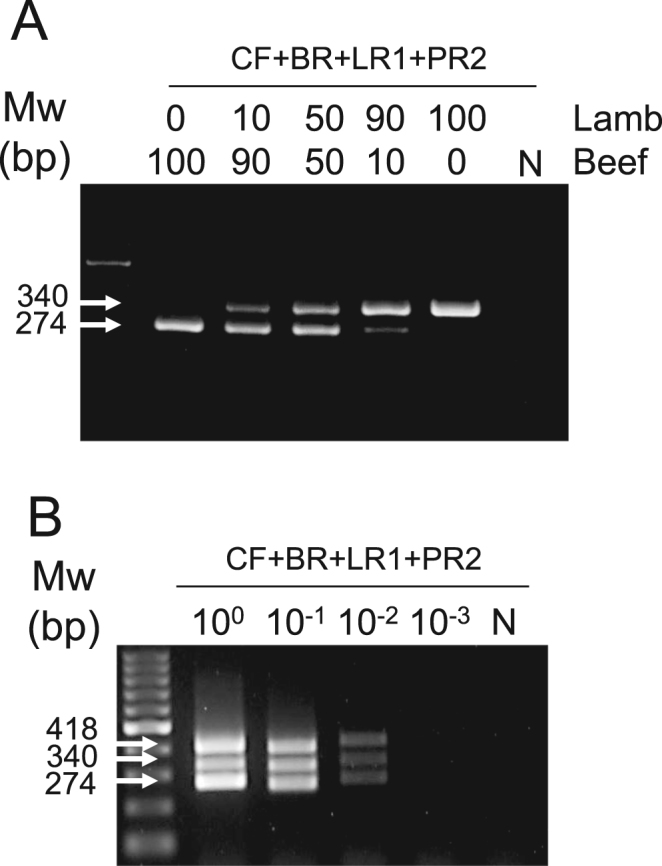
Direct-convection PCR of swabbed samples of mixed meats. (A) Swab samples were obtained from beef/lamb different ratio mixtures, and direct-convection PCR was performed. Gradual converse changes of band intensities were observed. (B) Swab samples were obtained from a 1:1:1 mixture of beef, lamb, and pork, and direct-convection PCR was performed. N, no template control; Mw, molecular weight marker.

The primers used in this article are given in Table 1. Fig. 1 shows evident DNA amplification from direct-convection PCR of swab samples eluted in 1× Taq DNA polymerase buffer and phosphate buffered saline. Fig. 2 shows that 10 min incubation with proteinase K and 1 min of heat inactivation comprise a sufficient swabbed DNA sample treatment. Fig. 3 shows that the multiplex direct-convection PCR meat detection protocol developed herein is sensitive enough to detect meat species in mixed meat samples. In addition, the swabbed sample can be diluted 100 times with the detection sensitivity maintained.

**Table 1:** Primers used for meat identification.

Name	Sequence (5′→3′)	Tm (°C)	Primer pair	Amplicon size (bp)
Common F (CF)	GACCTCCCAGCTCCATCAAAC ATCTCATCTTGATGAAA	64.2		
Beef-R (BR)	CTAGAAAAGTGTAAGACCCGTAATATAAG	53.2	CF/BR	274
Lamb-R1 (LR1)	AAACATAGCCTATGAATGCTGTGGCTATTGTC	60.3	CF/LR1	340
Pork-R2 (PR2)	CTGTTCCGATATAAGGGATAGCTGATAGTAGA	58	CF/PR2	418

## 2 Experimental design, materials, and methods

### 2.1 Materials

Meat samples (beef, lamb, and pork) were purchased from commercial sources in Korean markets.

### 2.2 DNA swab sample preparation

DNA swab samples were acquired as described in [1]. Briefly, a moistened swab was used to rub the surface of each meat piece or mixed meat samples, and transferred to a 1.5 mL tube containing 300 μL of PBS (10 mM Na_2_HPO_4_, 2.7 mM KH_2_PO_4_, 137 mM NaCl, and 2.7 mM KCl, pH 7.4), and 1× *PalmTaq* High-Speed buffer containing 1.5 mM MgCl_2_ (Ahram Biosystems, Inc., Korea), or water (Sigma, USA). Then, 0.1 volume of proteinase K (2–20 mg/mL) was added, and the samples were incubated at 56 °C for 10–30 min. This was followed by boiling for 1–5 min. Sample aliquots (1 μL) were used as DNA templates in subsequent PCR reactions. These experiments were performed in a Palm PCR G2-12 device (Ahram Biosystems) using the isothermal incubation setting.

### 2.3 Species-specific primer design and convection PCR

Primers were designed as described by Matsunaga et al. [2], with modification for convection PCR (Table 1). See [1] for detailed information. Convection PCR was performed using a Palm PCR device (G2-12, Ahram Biosystems) as described by the supplier. See [1] for detailed information. The speed level of convection PCR was set to F3, the annealing temperature was set to 60 °C, and PCR was performed over 30 cycles for 24 min, unless stated otherwise. After the completion of PCR, an aliquot of the PCR solution was analyzed by agarose gel electrophoresis. PCR products were visualized by fluorescence after ethidium bromide staining, and quantified with a densitometer (Ultra-Lum Imaging System, USA). All experiments were performed at least in triplicate.
