# Supplementary material for: Data for the optimization of conditions for meat species identification using ultra-fast multiplex direct-convection PCR
Source: Data Brief. 2017 Nov 4;16:15–8. doi: 10.1016/j.dib.2017.11.004 (PMC5686462; doi:10.1016/j.dib.2017.11.004)
Supplement: Supplementary file 1 — Supplementary material [file mmc1.pdf]

Conflict of Interest statement

A conflict of interest may exist when an author has a financial or other relationship with other people or organizations that may inappropriately influence the author's work. A conflict can be actual or potential and full disclosure to the Journal is the safest course. All submissions to the Journal must include disclosure of all relationships that could be viewed as presenting a potential conflict of interest. The Journal may use such information as a basis for editorial decisions and may publish such disclosures if they are believed to be important to readers in judging the manuscript. A decision may be made by the Journal not to publish on the basis of the declared conflict.

Under what circumstances must I disclose information about my working relationships?
At the end of the text, under a subheading "Conflicts of Interest", all authors must disclose any actual or potential conflict of interest including any financial, personal or other relationships with other people or organizations within three (3) years of beginning the work submitted that could inappropriately influence (bias) their work. Examples of potential conflicts of interest which should be disclosed include employment, consultancies, stock ownership, honoraria, paid expert testimony, patent applications/registrations, and grants or other funding.

Please detail below any such conflicts of interest or potential conflicts or state that no such conflicts exist:

No conflicts of interest exist.

What is the role of my funding source?
If funding has been provided, all sources of funding must be declared. This declaration should be made in an Acknowledgements section and placed before the References. Authors must describe the role of the study sponsor(s), if any, in study design; in the collection, analysis, and interpretation of data; in the writing of the report; and in the decision to submit the paper for publication.

The corresponding author should sign this declaration on behalf of all the authors:

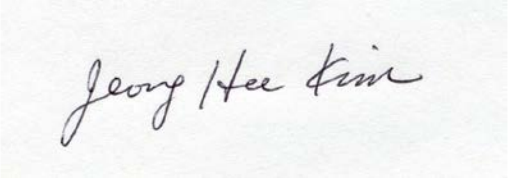

____
